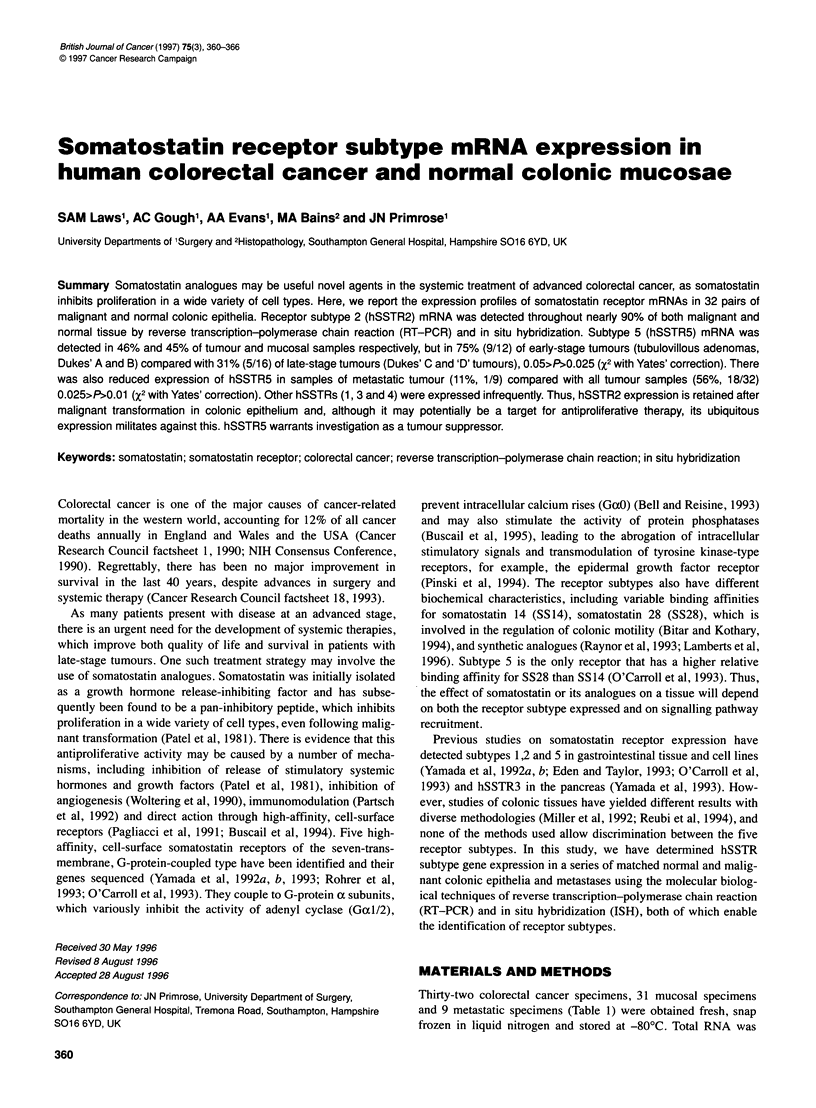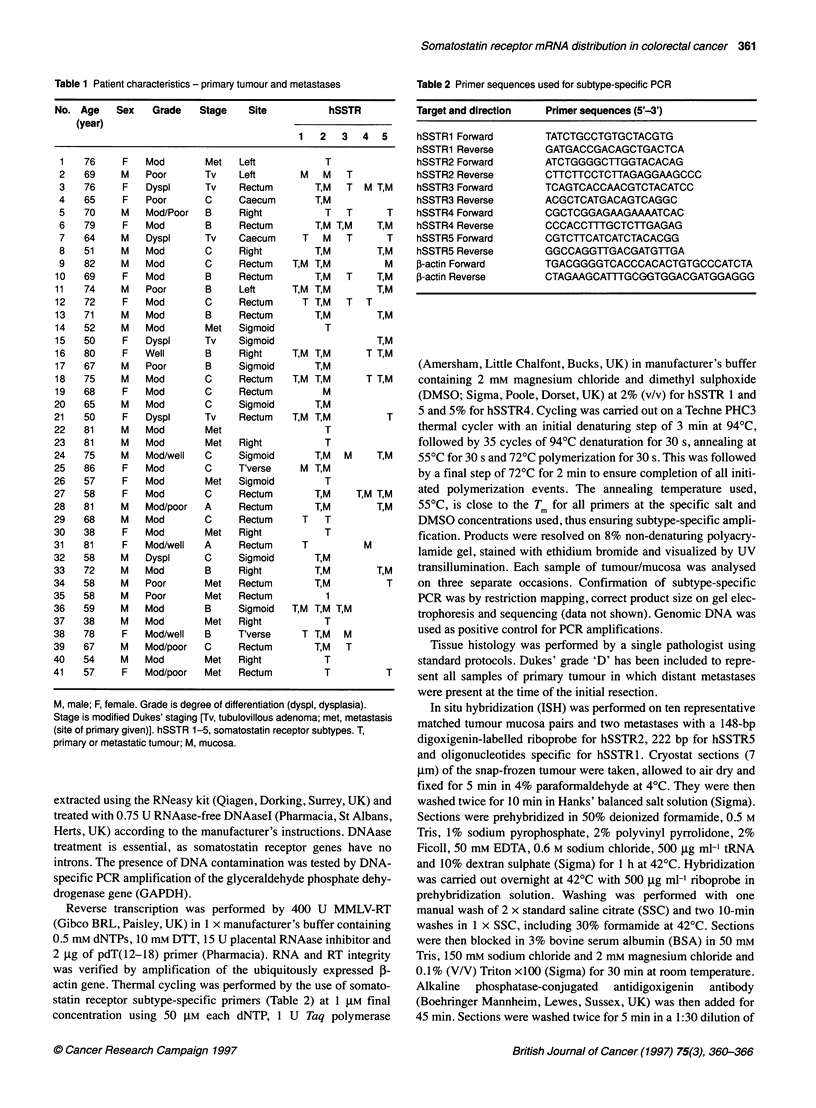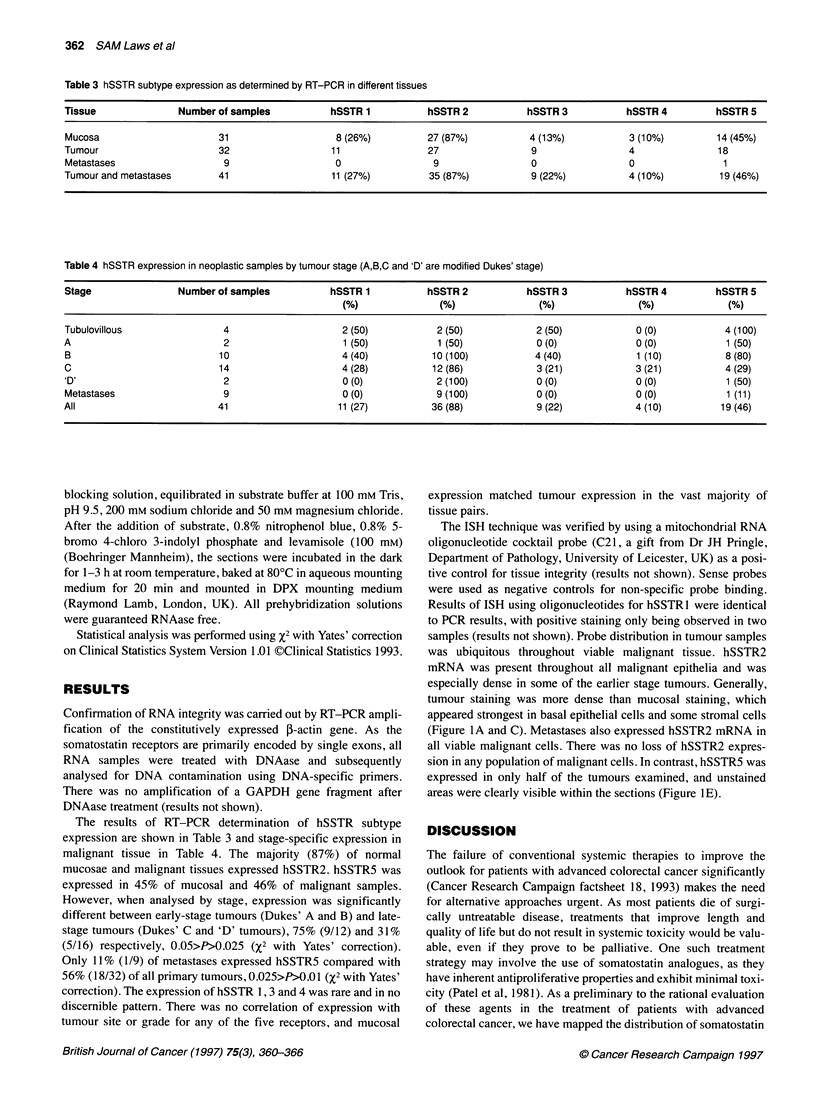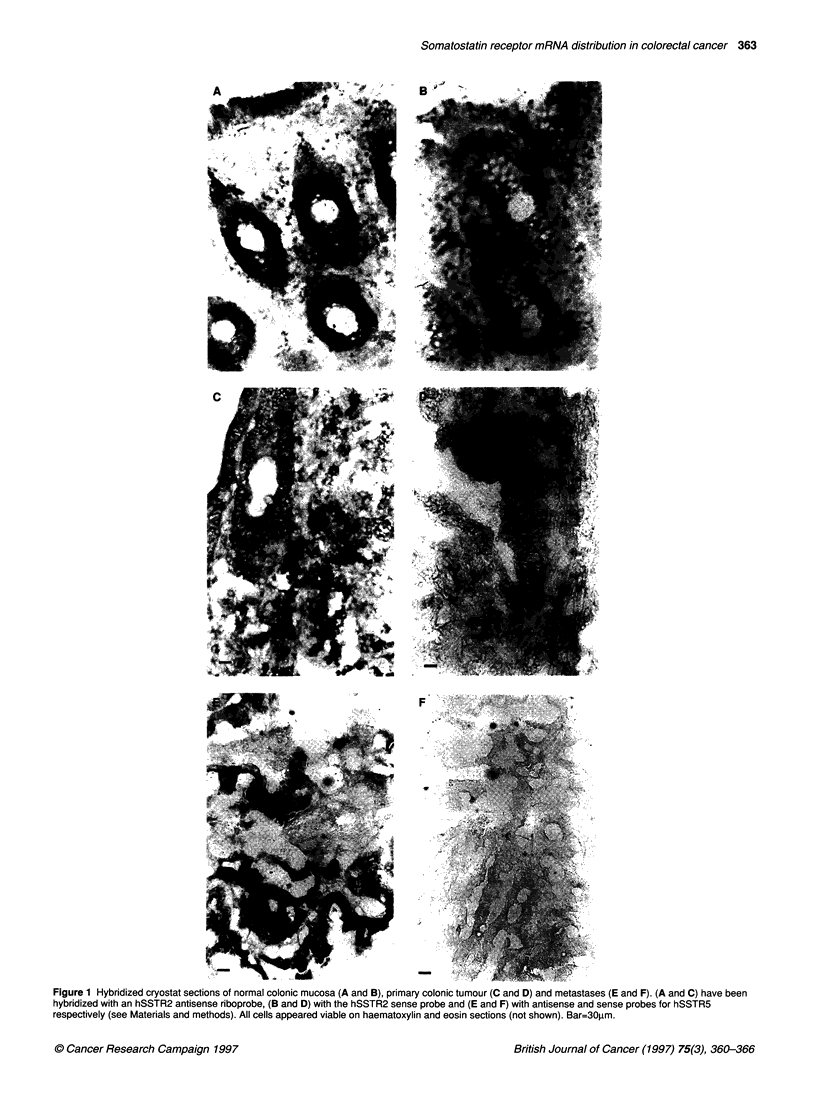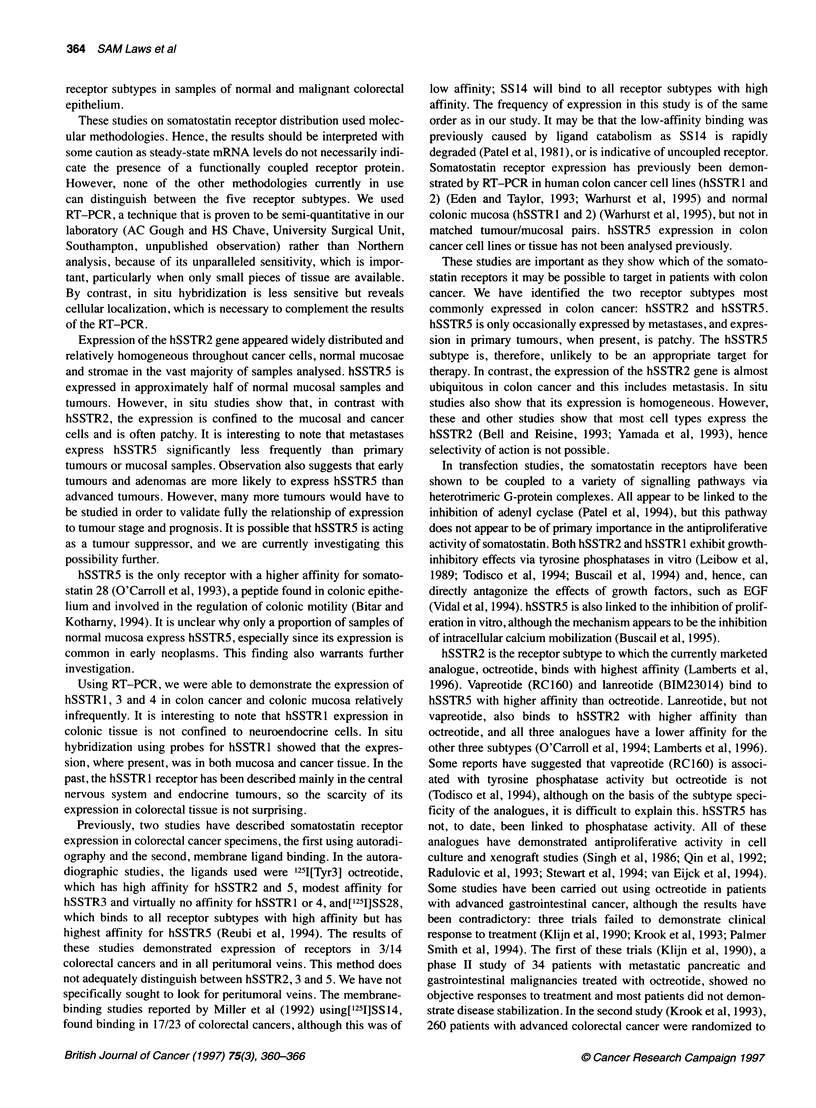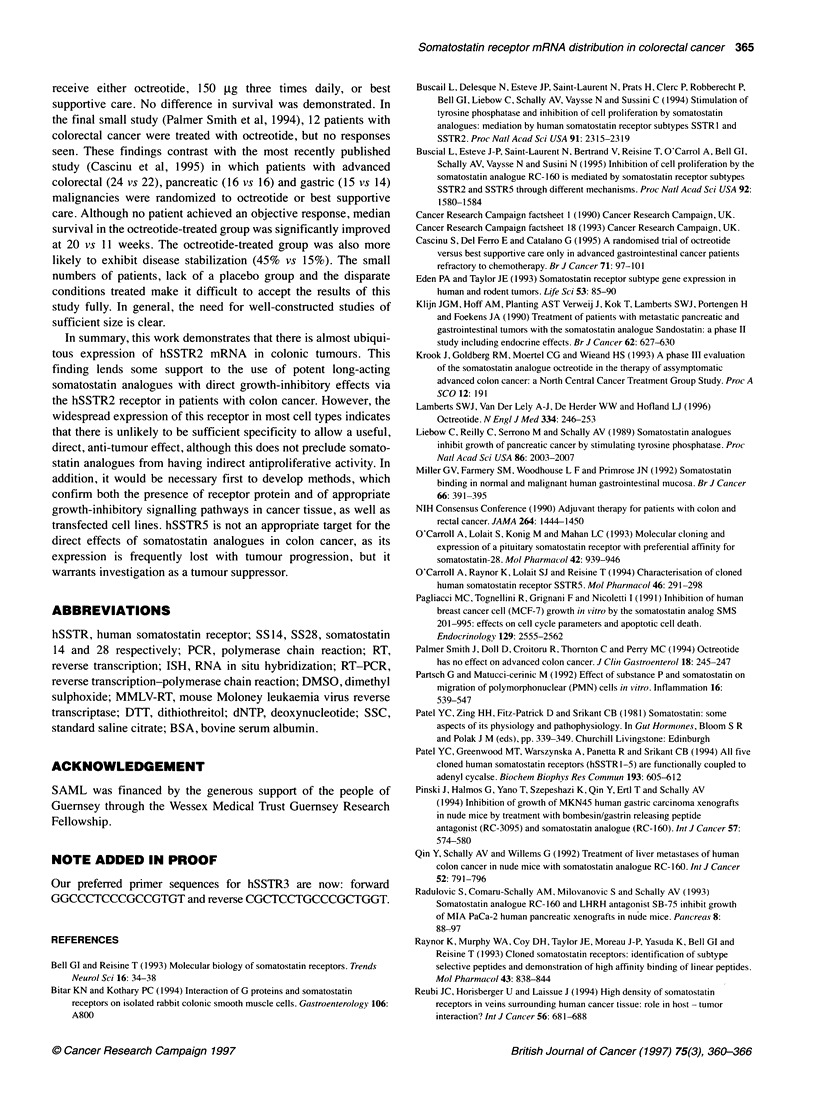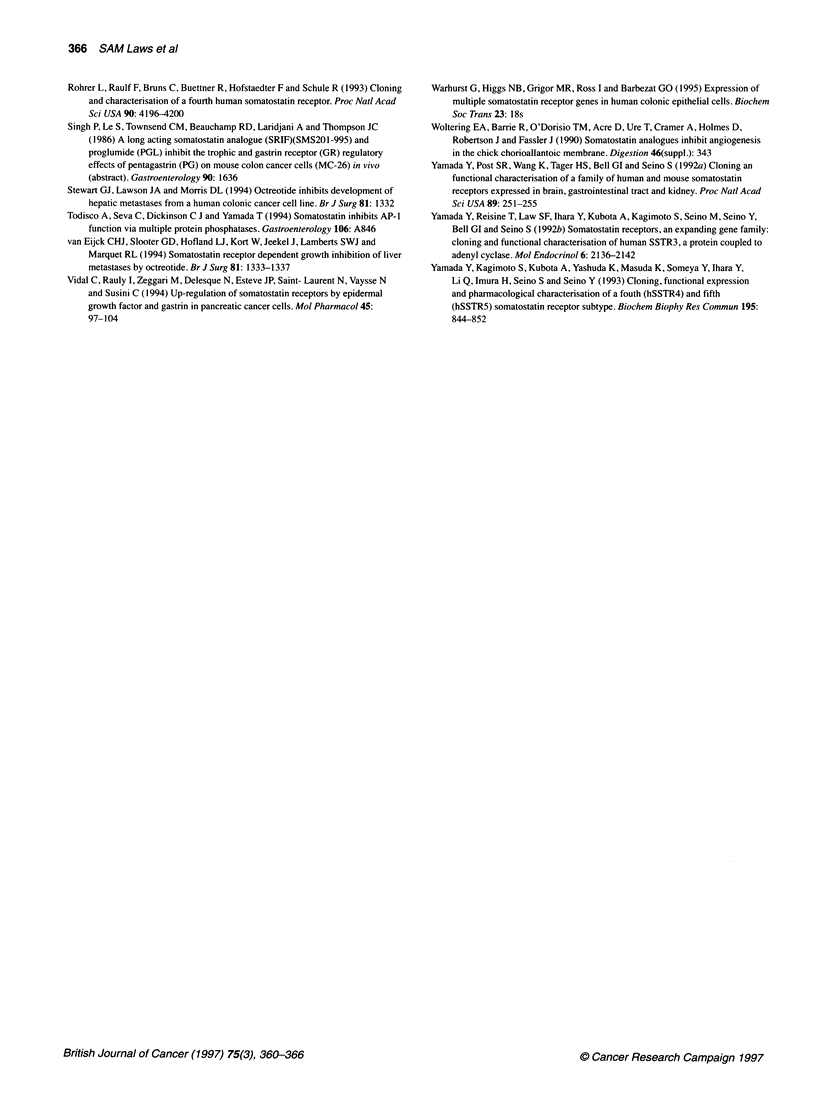# Somatostatin receptor subtype mRNA expression in human colorectal cancer and normal colonic mucosae.

**DOI:** 10.1038/bjc.1997.59

**Published:** 1997

**Authors:** S. A. Laws, A. C. Gough, A. A. Evans, M. A. Bains, J. N. Primrose

**Affiliations:** University Department of Surgery, Southampton General Hospital, Hampshire, UK.

## Abstract

**Images:**


British Journal of Cancer (1997) 75(3), 360–366
© 1997 Cancer Research Campaign

### Somatostatin receptor subtype mRNA expression in human colorectal cancer and normal colonic mucosae

**SAM Laws[1], AC Gough[1], AA Evans[1], MA Bains[2] and JN Primrose[1]**

University Departments of [1]Surgery and [2]Histopathology, Southampton General Hospital, Hampshire SO16 6YD, UK

**Summary** Somatostatin analogues may be useful novel agents in the systemic treatment of advanced colorectal cancer, as somatostatin inhibits proliferation in a wide variety of cell types. Here, we report the expression profiles of somatostatin receptor mRNAs in 32 pairs of malignant and normal colonic epithelia. Receptor subtype 2 (hSSTR2) mRNA was detected throughout nearly 90% of both malignant and normal tissue by reverse transcription–polymerase chain reaction (RT–PCR) and in situ hybridization. Subtype 5 (hSSTR5) mRNA was detected in 46% and 45% of tumour and mucosal samples respectively, but in 75% (9/12) of early-stage tumours (tubulovillous adenomas, Dukes' A and B) compared with 31% (5/16) of late-stage tumours (Dukes' C and 'D' tumours), 0.05>$P$>0.025 ($\chi^2$ with Yates' correction). There was also reduced expression of hSSTR5 in samples of metastatic tumour (11%, 1/9) compared with all tumour samples (56%, 18/32) 0.025>$P$>0.01 ($\chi^2$ with Yates' correction). Other hSSTRs (1, 3 and 4) were expressed infrequently. Thus, hSSTR2 expression is retained after malignant transformation in colonic epithelium and, although it may potentially be a target for antiproliferative therapy, its ubiquitous expression militates against this. hSSTR5 warrants investigation as a tumour suppressor.

**Keywords:** somatostatin; somatostatin receptor; colorectal cancer; reverse transcription–polymerase chain reaction; in situ hybridization

Colorectal cancer is one of the major causes of cancer-related mortality in the western world, accounting for 12% of all cancer deaths annually in England and Wales and the USA (Cancer Research Council factsheet 1, 1990; NIH Consensus Conference, 1990). Regrettably, there has been no major improvement in survival in the last 40 years, despite advances in surgery and systemic therapy (Cancer Research Council factsheet 18, 1993).

As many patients present with disease at an advanced stage, there is an urgent need for the development of systemic therapies, which improve both quality of life and survival in patients with late-stage tumours. One such treatment strategy may involve the use of somatostatin analogues. Somatostatin was initially isolated as a growth hormone release-inhibiting factor and has subsequently been found to be a pan-inhibitory peptide, which inhibits proliferation in a wide variety of cell types, even following malignant transformation (Patel et al, 1981). There is evidence that this antiproliferative activity may be caused by a number of mechanisms, including inhibition of release of stimulatory systemic hormones and growth factors (Patel et al, 1981), inhibition of angiogenesis (Woltering et al, 1990), immunomodulation (Partsch et al, 1992) and direct action through high-affinity, cell-surface receptors (Pagliacci et al, 1991; Buscail et al, 1994). Five high-affinity, cell-surface somatostatin receptors of the seven-transmembrane, G-protein-coupled type have been identified and their genes sequenced (Yamada et al, 1992*a*, *b*, 1993; Rohrer et al, 1993; O'Carroll et al, 1993). They couple to G-protein α subunits, which variously inhibit the activity of adenyl cyclase (Gαl/2), prevent intracellular calcium rises (Gα0) (Bell and Reisine, 1993) and may also stimulate the activity of protein phosphatases (Buscail et al, 1995), leading to the abrogation of intracellular stimulatory signals and transmodulation of tyrosine kinase-type receptors, for example, the epidermal growth factor receptor (Pinski et al, 1994). The receptor subtypes also have different biochemical characteristics, including variable binding affinities for somatostatin 14 (SS14), somatostatin 28 (SS28), which is involved in the regulation of colonic motility (Bitar and Kothary, 1994), and synthetic analogues (Raynor et al, 1993; Lamberts et al, 1996). Subtype 5 is the only receptor that has a higher relative binding affinity for SS28 than SS14 (O'Carroll et al, 1993). Thus, the effect of somatostatin or its analogues on a tissue will depend on both the receptor subtype expressed and on signalling pathway recruitment.

Previous studies on somatostatin receptor expression have detected subtypes 1,2 and 5 in gastrointestinal tissue and cell lines (Yamada et al, 1992*a*, *b*; Eden and Taylor, 1993; O'Carroll et al, 1993) and hSSTR3 in the pancreas (Yamada et al, 1993). However, studies of colonic tissues have yielded different results with diverse methodologies (Miller et al, 1992; Reubi et al, 1994), and none of the methods used allow discrimination between the five receptor subtypes. In this study, we have determined hSSTR subtype gene expression in a series of matched normal and malignant colonic epithelia and metastases using the molecular biological techniques of reverse transcription–polymerase chain reaction (RT–PCR) and in situ hybridization (ISH), both of which enable the identification of receptor subtypes.

*Received 30 May 1996*
*Revised 8 August 1996*
*Accepted 28 August 1996*

*Correspondence to:* JN Primrose, University Department of Surgery, Southampton General Hospital, Tremona Road, Southampton, Hampshire SO16 6YD, UK

### MATERIALS AND METHODS

Thirty-two colorectal cancer specimens, 31 mucosal specimens and 9 metastatic specimens (Table 1) were obtained fresh, snap frozen in liquid nitrogen and stored at –80°C. Total RNA was

**Table 1** Patient characteristics – primary tumour and metastases

| No. | Age (year) | Sex | Grade | Stage | Site | hSSTR | | | | |
|---|---|---|---|---|---|---|---|---|---|---|
| | | | | | | 1 | 2 | 3 | 4 | 5 |
| 1 | 76 | F | Mod | Met | Left | | T | | | |
| 2 | 69 | M | Poor | Tv | Left | M | M | T | | |
| 3 | 76 | F | Dyspl | Tv | Rectum | | T,M | T | M | T,M |
| 4 | 65 | F | Poor | C | Caecum | | T,M | | | |
| 5 | 70 | M | Mod/Poor | B | Right | | T | T | | T |
| 6 | 79 | F | Mod | B | Rectum | | T,M | T,M | | T,M |
| 7 | 64 | M | Dyspl | Tv | Caecum | T | M | T | | T |
| 8 | 51 | M | Mod | C | Right | | T,M | | | T,M |
| 9 | 82 | M | Mod | C | Rectum | T,M | T,M | | | M |
| 10 | 69 | F | Mod | B | Rectum | | T,M | T | | T,M |
| 11 | 74 | M | Poor | B | Left | T,M | T,M | | | T,M |
| 12 | 72 | F | Mod | C | Rectum | T | T,M | T | T | |
| 13 | 71 | M | Mod | B | Rectum | | T,M | | | T,M |
| 14 | 52 | M | Mod | Met | Sigmoid | | T | | | |
| 15 | 50 | F | Dyspl | Tv | Sigmoid | | | | | T,M |
| 16 | 80 | F | Well | B | Right | T,M | T,M | | T | T,M |
| 17 | 67 | M | Poor | B | Sigmoid | | T,M | | | |
| 18 | 75 | M | Mod | C | Rectum | T,M | T,M | | T | T,M |
| 19 | 68 | F | Mod | C | Rectum | | M | | | |
| 20 | 65 | M | Mod | C | Sigmoid | | T,M | | | |
| 21 | 50 | F | Dyspl | Tv | Rectum | T,M | T,M | | | T |
| 22 | 81 | M | Mod | Met | | | T | | | |
| 23 | 81 | M | Mod | Met | Right | | T | | | |
| 24 | 75 | M | Mod/well | C | Sigmoid | | T,M | M | | T,M |
| 25 | 86 | F | Mod | C | T'verse | M | T,M | | | |
| 26 | 57 | F | Mod | Met | Sigmoid | | T | | | |
| 27 | 58 | F | Mod | C | Rectum | | T,M | | T,M | T,M |
| 28 | 81 | M | Mod/poor | A | Rectum | | T,M | | | T,M |
| 29 | 68 | M | Mod | C | Rectum | T | T | | | |
| 30 | 38 | F | Mod | Met | Right | | T | | | |
| 31 | 81 | F | Mod/well | A | Rectum | T | | | M | |
| 32 | 58 | M | Dyspl | C | Sigmoid | | T,M | | | |
| 33 | 72 | M | Mod | B | Right | | T,M | | | T,M |
| 34 | 58 | M | Poor | Met | Rectum | | T,M | | | T |
| 35 | 58 | M | Poor | Met | Rectum | | 1 | | | |
| 36 | 59 | M | Mod | B | Sigmoid | T,M | T,M | T,M | | |
| 37 | 38 | M | Mod | Met | Right | | T | | | |
| 38 | 78 | F | Mod/well | B | T'verse | T | T,M | M | | |
| 39 | 67 | M | Mod/poor | C | Rectum | | T,M | T | | |
| 40 | 54 | M | Mod | Met | Right | | T | | | |
| 41 | 57 | F | Mod/poor | Met | Rectum | | T | | | T |

M, male; F, female. Grade is degree of differentiation (dyspl, dysplasia). Stage is modified Dukes' staging [Tv, tubulovillous adenoma; met, metastasis (site of primary given)]. hSSTR 1–5, somatostatin receptor subtypes. T, primary or metastatic tumour; M, mucosa.

**Table 2** Primer sequences used for subtype-specific PCR

| Target and direction | Primer sequences (5'–3') |
|---|---|
| hSSTR1 Forward | TATCTGCCTGTGCTACGTG |
| hSSTR1 Reverse | GATGACCGACAGCTGACTCA |
| hSSTR2 Forward | ATCTGGGGCTTGGTACACAG |
| hSSTR2 Reverse | CTTCTTCCTCTTAGAGGAAGCCC |
| hSSTR3 Forward | TCAGTCACCAACGTCTACATCC |
| hSSTR3 Reverse | ACGCTCATGACAGTCAGGC |
| hSSTR4 Forward | CGCTCGGAGAAGAAAATCAC |
| hSSTR4 Reverse | CCCACCTTTGCTCTTGAGAG |
| hSSTR5 Forward | CGTCTTCATCATCTACACGG |
| hSSTR5 Reverse | GGCCAGGTTGACGATGTTGA |
| β-actin Forward | TGACGGGGTCACCCACACTGTGCCCATCTA |
| β-actin Reverse | CTAGAAGCATTTGCGGTGGACGATGGAGGG |

extracted using the RNeasy kit (Qiagen, Dorking, Surrey, UK) and treated with 0.75 U RNAase-free DNAaseI (Pharmacia, St Albans, Herts, UK) according to the manufacturer's instructions. DNAase treatment is essential, as somatostatin receptor genes have no introns. The presence of DNA contamination was tested by DNA-specific PCR amplification of the glyceraldehyde phosphate dehydrogenase gene (GAPDH).

Reverse transcription was performed by 400 U MMLV-RT (Gibco BRL, Paisley, UK) in 1 × manufacturer's buffer containing 0.5 mM dNTPs, 10 mM DTT, 15 U placental RNAase inhibitor and 2 μg of pdT(12–18) primer (Pharmacia). RNA and RT integrity was verified by amplification of the ubiquitously expressed β-actin gene. Thermal cycling was performed by the use of somatostatin receptor subtype-specific primers (Table 2) at 1 μM final concentration using 50 μM each dNTP, 1 U *Taq* polymerase (Amersham, Little Chalfont, Bucks, UK) in manufacturer's buffer containing 2 mM magnesium chloride and dimethyl sulphoxide (DMSO; Sigma, Poole, Dorset, UK) at 2% (v/v) for hSSTR 1 and 5 and 5% for hSSTR4. Cycling was carried out on a Techne PHC3 thermal cycler with an initial denaturing step of 3 min at 94°C, followed by 35 cycles of 94°C denaturation for 30 s, annealing at 55°C for 30 s and 72°C polymerization for 30 s. This was followed by a final step of 72°C for 2 min to ensure completion of all initiated polymerization events. The annealing temperature used, 55°C, is close to the $T_m$ for all primers at the specific salt and DMSO concentrations used, thus ensuring subtype-specific amplification. Products were resolved on 8% non-denaturing polyacrylamide gel, stained with ethidium bromide and visualized by UV transillumination. Each sample of tumour/mucosa was analysed on three separate occasions. Confirmation of subtype-specific PCR was by restriction mapping, correct product size on gel electrophoresis and sequencing (data not shown). Genomic DNA was used as positive control for PCR amplifications.

Tissue histology was performed by a single pathologist using standard protocols. Dukes' grade 'D' has been included to represent all samples of primary tumour in which distant metastases were present at the time of the initial resection.

In situ hybridization (ISH) was performed on ten representative matched tumour mucosa pairs and two metastases with a 148-bp digoxigenin-labelled riboprobe for hSSTR2, 222 bp for hSSTR5 and oligonucleotides specific for hSSTR1. Cryostat sections (7 μm) of the snap-frozen tumour were taken, allowed to air dry and fixed for 5 min in 4% paraformaldehyde at 4°C. They were then washed twice for 10 min in Hanks' balanced salt solution (Sigma). Sections were prehybridized in 50% deionized formamide, 0.5 M Tris, 1% sodium pyrophosphate, 2% polyvinyl pyrrolidone, 2% Ficoll, 50 mM EDTA, 0.6 M sodium chloride, 500 μg ml$^{-1}$ tRNA and 10% dextran sulphate (Sigma) for 1 h at 42°C. Hybridization was carried out overnight at 42°C with 500 μg ml$^{-1}$ riboprobe in prehybridization solution. Washing was performed with one manual wash of 2 × standard saline citrate (SSC) and two 10-min washes in 1 × SSC, including 30% formamide at 42°C. Sections were then blocked in 3% bovine serum albumin (BSA) in 50 mM Tris, 150 mM sodium chloride and 2 mM magnesium chloride and 0.1% (V/V) Triton ×100 (Sigma) for 30 min at room temperature. Alkaline phosphatase-conjugated antidigoxigenin antibody (Boehringer Mannheim, Lewes, Sussex, UK) was then added for 45 min. Sections were washed twice for 5 min in a 1:30 dilution of

© Cancer Research Campaign 1997

**Table 3** hSSTR subtype expression as determined by RT–PCR in different tissues

| Tissue | Number of samples | hSSTR 1 | hSSTR 2 | hSSTR 3 | hSSTR 4 | hSSTR 5 |
|---|---|---|---|---|---|---|
| Mucosa | 31 | 8 (26%) | 27 (87%) | 4 (13%) | 3 (10%) | 14 (45%) |
| Tumour | 32 | 11 | 27 | 9 | 4 | 18 |
| Metastases | 9 | 0 | 9 | 0 | 0 | 1 |
| Tumour and metastases | 41 | 11 (27%) | 35 (87%) | 9 (22%) | 4 (10%) | 19 (46%) |

**Table 4** hSSTR expression in neoplastic samples by tumour stage (A,B,C and 'D' are modified Dukes' stage)

| Stage | Number of samples | hSSTR 1 (%) | hSSTR 2 (%) | hSSTR 3 (%) | hSSTR 4 (%) | hSSTR 5 (%) |
|---|---|---|---|---|---|---|
| Tubulovillous | 4 | 2 (50) | 2 (50) | 2 (50) | 0 (0) | 4 (100) |
| A | 2 | 1 (50) | 1 (50) | 0 (0) | 0 (0) | 1 (50) |
| B | 10 | 4 (40) | 10 (100) | 4 (40) | 1 (10) | 8 (80) |
| C | 14 | 4 (28) | 12 (86) | 3 (21) | 3 (21) | 4 (29) |
| 'D' | 2 | 0 (0) | 2 (100) | 0 (0) | 0 (0) | 1 (50) |
| Metastases | 9 | 0 (0) | 9 (100) | 0 (0) | 0 (0) | 1 (11) |
| All | 41 | 11 (27) | 36 (88) | 9 (22) | 4 (10) | 19 (46) |

blocking solution, equilibrated in substrate buffer at 100 mM Tris, pH 9.5, 200 mM sodium chloride and 50 mM magnesium chloride. After the addition of substrate, 0.8% nitrophenol blue, 0.8% 5-bromo 4-chloro 3-indolyl phosphate and levamisole (100 mM) (Boehringer Mannheim), the sections were incubated in the dark for 1–3 h at room temperature, baked at 80°C in aqueous mounting medium for 20 min and mounted in DPX mounting medium (Raymond Lamb, London, UK). All prehybridization solutions were guaranteed RNAase free.

Statistical analysis was performed using $\chi^2$ with Yates' correction on Clinical Statistics System Version 1.01 ©Clinical Statistics 1993.

### RESULTS

Confirmation of RNA integrity was carried out by RT–PCR amplification of the constitutively expressed β-actin gene. As the somatostatin receptors are primarily encoded by single exons, all RNA samples were treated with DNAase and subsequently analysed for DNA contamination using DNA-specific primers. There was no amplification of a GAPDH gene fragment after DNAase treatment (results not shown).

The results of RT–PCR determination of hSSTR subtype expression are shown in Table 3 and stage-specific expression in malignant tissue in Table 4. The majority (87%) of normal mucosae and malignant tissues expressed hSSTR2. hSSTR5 was expressed in 45% of mucosal and 46% of malignant samples. However, when analysed by stage, expression was significantly different between early-stage tumours (Dukes' A and B) and late-stage tumours (Dukes' C and 'D' tumours), 75% (9/12) and 31% (5/16) respectively, $0.05>P>0.025$ ($\chi^2$ with Yates' correction). Only 11% (1/9) of metastases expressed hSSTR5 compared with 56% (18/32) of all primary tumours, $0.025>P>0.01$ ($\chi^2$ with Yates' correction). The expression of hSSTR 1, 3 and 4 was rare and in no discernible pattern. There was no correlation of expression with tumour site or grade for any of the five receptors, and mucosal expression matched tumour expression in the vast majority of tissue pairs.

The ISH technique was verified by using a mitochondrial RNA oligonucleotide cocktail probe (C21, a gift from Dr JH Pringle, Department of Pathology, University of Leicester, UK) as a positive control for tissue integrity (results not shown). Sense probes were used as negative controls for non-specific probe binding. Results of ISH using oligonucleotides for hSSTR1 were identical to PCR results, with positive staining only being observed in two samples (results not shown). Probe distribution in tumour samples was ubiquitous throughout viable malignant tissue. hSSTR2 mRNA was present throughout all malignant epithelia and was especially dense in some of the earlier stage tumours. Generally, tumour staining was more dense than mucosal staining, which appeared strongest in basal epithelial cells and some stromal cells (Figure 1A and C). Metastases also expressed hSSTR2 mRNA in all viable malignant cells. There was no loss of hSSTR2 expression in any population of malignant cells. In contrast, hSSTR5 was expressed in only half of the tumours examined, and unstained areas were clearly visible within the sections (Figure 1E).

### DISCUSSION

The failure of conventional systemic therapies to improve the outlook for patients with advanced colorectal cancer significantly (Cancer Research Campaign factsheet 18, 1993) makes the need for alternative approaches urgent. As most patients die of surgically untreatable disease, treatments that improve length and quality of life but do not result in systemic toxicity would be valuable, even if they prove to be palliative. One such treatment strategy may involve the use of somatostatin analogues, as they have inherent antiproliferative properties and exhibit minimal toxicity (Patel et al, 1981). As a preliminary to the rational evaluation of these agents in the treatment of patients with advanced colorectal cancer, we have mapped the distribution of somatostatin

© Cancer Research Campaign 1997

**Figure 1** Hybridized cryostat sections of normal colonic mucosa (**A** and **B**), primary colonic tumour (**C** and **D**) and metastases (**E** and **F**). (**A** and **C**) have been hybridized with an hSSTR2 antisense riboprobe, (**B** and **D**) with the hSSTR2 sense probe and (**E** and **F**) with antisense and sense probes for hSSTR5 respectively (see Materials and methods). All cells appeared viable on haematoxylin and eosin sections (not shown). Bar=30μm.

receptor subtypes in samples of normal and malignant colorectal epithelium.

These studies on somatostatin receptor distribution used molecular methodologies. Hence, the results should be interpreted with some caution as steady-state mRNA levels do not necessarily indicate the presence of a functionally coupled receptor protein. However, none of the other methodologies currently in use can distinguish between the five receptor subtypes. We used RT–PCR, a technique that is proven to be semi-quantitative in our laboratory (AC Gough and HS Chave, University Surgical Unit, Southampton, unpublished observation) rather than Northern analysis, because of its unparalleled sensitivity, which is important, particularly when only small pieces of tissue are available. By contrast, in situ hybridization is less sensitive but reveals cellular localization, which is necessary to complement the results of the RT–PCR.

Expression of the hSSTR2 gene appeared widely distributed and relatively homogeneous throughout cancer cells, normal mucosae and stromae in the vast majority of samples analysed. hSSTR5 is expressed in approximately half of normal mucosal samples and tumours. However, in situ studies show that, in contrast with hSSTR2, the expression is confined to the mucosal and cancer cells and is often patchy. It is interesting to note that metastases express hSSTR5 significantly less frequently than primary tumours or mucosal samples. Observation also suggests that early tumours and adenomas are more likely to express hSSTR5 than advanced tumours. However, many more tumours would have to be studied in order to validate fully the relationship of expression to tumour stage and prognosis. It is possible that hSSTR5 is acting as a tumour suppressor, and we are currently investigating this possibility further.

hSSTR5 is the only receptor with a higher affinity for somatostatin 28 (O'Carroll et al, 1993), a peptide found in colonic epithelium and involved in the regulation of colonic motility (Bitar and Kotharny, 1994). It is unclear why only a proportion of samples of normal mucosa express hSSTR5, especially since its expression is common in early neoplasms. This finding also warrants further investigation.

Using RT–PCR, we were able to demonstrate the expression of hSSTR1, 3 and 4 in colon cancer and colonic mucosa relatively infrequently. It is interesting to note that hSSTR1 expression in colonic tissue is not confined to neuroendocrine cells. In situ hybridization using probes for hSSTR1 showed that the expression, where present, was in both mucosa and cancer tissue. In the past, the hSSTR1 receptor has been described mainly in the central nervous system and endocrine tumours, so the scarcity of its expression in colorectal tissue is not surprising.

Previously, two studies have described somatostatin receptor expression in colorectal cancer specimens, the first using autoradiography and the second, membrane ligand binding. In the autoradiographic studies, the ligands used were $^{125}$I[Tyr3] octreotide, which has high affinity for hSSTR2 and 5, modest affinity for hSSTR3 and virtually no affinity for hSSTR1 or 4, and[$^{125}$I]SS28, which binds to all receptor subtypes with high affinity but has highest affinity for hSSTR5 (Reubi et al, 1994). The results of these studies demonstrated expression of receptors in 3/14 colorectal cancers and in all peritumoral veins. This method does not adequately distinguish between hSSTR2, 3 and 5. We have not specifically sought to look for peritumoral veins. The membrane-binding studies reported by Miller et al (1992) using[$^{125}$I]SS14, found binding in 17/23 of colorectal cancers, although this was of low affinity; SS14 will bind to all receptor subtypes with high affinity. The frequency of expression in this study is of the same order as in our study. It may be that the low-affinity binding was previously caused by ligand catabolism as SS14 is rapidly degraded (Patel et al, 1981), or is indicative of uncoupled receptor. Somatostatin receptor expression has previously been demonstrated by RT–PCR in human colon cancer cell lines (hSSTR1 and 2) (Eden and Taylor, 1993; Warhurst et al, 1995) and normal colonic mucosa (hSSTR1 and 2) (Warhurst et al, 1995), but not in matched tumour/mucosal pairs. hSSTR5 expression in colon cancer cell lines or tissue has not been analysed previously.

These studies are important as they show which of the somatostatin receptors it may be possible to target in patients with colon cancer. We have identified the two receptor subtypes most commonly expressed in colon cancer: hSSTR2 and hSSTR5. hSSTR5 is only occasionally expressed by metastases, and expression in primary tumours, when present, is patchy. The hSSTR5 subtype is, therefore, unlikely to be an appropriate target for therapy. In contrast, the expression of the hSSTR2 gene is almost ubiquitous in colon cancer and this includes metastasis. In situ studies also show that its expression is homogeneous. However, these and other studies show that most cell types express the hSSTR2 (Bell and Reisine, 1993; Yamada et al, 1993), hence selectivity of action is not possible.

In transfection studies, the somatostatin receptors have been shown to be coupled to a variety of signalling pathways via heterotrimeric G-protein complexes. All appear to be linked to the inhibition of adenyl cyclase (Patel et al, 1994), but this pathway does not appear to be of primary importance in the antiproliferative activity of somatostatin. Both hSSTR2 and hSSTR1 exhibit growth-inhibitory effects via tyrosine phosphatases in vitro (Leibow et al, 1989; Todisco et al, 1994; Buscail et al, 1994) and, hence, can directly antagonize the effects of growth factors, such as EGF (Vidal et al, 1994). hSSTR5 is also linked to the inhibition of proliferation in vitro, although the mechanism appears to be the inhibition of intracellular calcium mobilization (Buscail et al, 1995).

hSSTR2 is the receptor subtype to which the currently marketed analogue, octreotide, binds with highest affinity (Lamberts et al, 1996). Vapreotide (RC160) and lanreotide (BIM23014) bind to hSSTR5 with higher affinity than octreotide. Lanreotide, but not vapreotide, also binds to hSSTR2 with higher affinity than octreotide, and all three analogues have a lower affinity for the other three subtypes (O'Carroll et al, 1994; Lamberts et al, 1996). Some reports have suggested that vapreotide (RC160) is associated with tyrosine phosphatase activity but octreotide is not (Todisco et al, 1994), although on the basis of the subtype specificity of the analogues, it is difficult to explain this. hSSTR5 has not, to date, been linked to phosphatase activity. All of these analogues have demonstrated antiproliferative activity in cell culture and xenograft studies (Singh et al, 1986; Qin et al, 1992; Radulovic et al, 1993; Stewart et al, 1994; van Eijck et al, 1994). Some studies have been carried out using octreotide in patients with advanced gastrointestinal cancer, although the results have been contradictory: three trials failed to demonstrate clinical response to treatment (Klijn et al, 1990; Krook et al, 1993; Palmer Smith et al, 1994). The first of these trials (Klijn et al, 1990), a phase II study of 34 patients with metastatic pancreatic and gastrointestinal malignancies treated with octreotide, showed no objective responses to treatment and most patients did not demonstrate disease stabilization. In the second study (Krook et al, 1993), 260 patients with advanced colorectal cancer were randomized to

 © Cancer Research Campaign 1997

receive either octreotide, 150 μg three times daily, or best supportive care. No difference in survival was demonstrated. In the final small study (Palmer Smith et al, 1994), 12 patients with colorectal cancer were treated with octreotide, but no responses seen. These findings contrast with the most recently published study (Cascinu et al, 1995) in which patients with advanced colorectal (24 *vs* 22), pancreatic (16 *vs* 16) and gastric (15 *vs* 14) malignancies were randomized to octreotide or best supportive care. Although no patient achieved an objective response, median survival in the octreotide-treated group was significantly improved at 20 *vs* 11 weeks. The octreotide-treated group was also more likely to exhibit disease stabilization (45% *vs* 15%). The small numbers of patients, lack of a placebo group and the disparate conditions treated make it difficult to accept the results of this study fully. In general, the need for well-constructed studies of sufficient size is clear.

In summary, this work demonstrates that there is almost ubiquitous expression of hSSTR2 mRNA in colonic tumours. This finding lends some support to the use of potent long-acting somatostatin analogues with direct growth-inhibitory effects via the hSSTR2 receptor in patients with colon cancer. However, the widespread expression of this receptor in most cell types indicates that there is unlikely to be sufficient specificity to allow a useful, direct, anti-tumour effect, although this does not preclude somatostatin analogues from having indirect antiproliferative activity. In addition, it would be necessary first to develop methods, which confirm both the presence of receptor protein and of appropriate growth-inhibitory signalling pathways in cancer tissue, as well as transfected cell lines. hSSTR5 is not an appropriate target for the direct effects of somatostatin analogues in colon cancer, as its expression is frequently lost with tumour progression, but it warrants investigation as a tumour suppressor.

### ABBREVIATIONS

hSSTR, human somatostatin receptor; SS14, SS28, somatostatin 14 and 28 respectively; PCR, polymerase chain reaction; RT, reverse transcription; ISH, RNA in situ hybridization; RT–PCR, reverse transcription–polymerase chain reaction; DMSO, dimethyl sulphoxide; MMLV-RT, mouse Moloney leukaemia virus reverse transcriptase; DTT, dithiothreitol; dNTP, deoxynucleotide; SSC, standard saline citrate; BSA, bovine serum albumin.

### ACKNOWLEDGEMENT

SAML was financed by the generous support of the people of Guernsey through the Wessex Medical Trust Guernsey Research Fellowship.

### NOTE ADDED IN PROOF

Our preferred primer sequences for hSSTR3 are now: forward GGCCCTCCCGCCGTGT and reverse CGCTCCTGCCCGCTGGT.

### REFERENCES

Bell GI and Reisine T (1993) Molecular biology of somatostatin receptors. *Trends Neurol Sci* **16**: 34–38

Bitar KN and Kothary PC (1994) Interaction of G proteins and somatostatin receptors on isolated rabbit colonic smooth muscle cells. *Gastroenterology* **106**: A800

Buscail L, Delesque N, Esteve JP, Saint-Laurent N, Prats H, Clerc P, Robberecht P, Bell GI, Liebow C, Schally AV, Vaysse N and Sussini C (1994) Stimulation of tyrosine phosphatase and inhibition of cell proliferation by somatostatin analogues: mediation by human somatostatin receptor subtypes SSTR1 and SSTR2. *Proc Natl Acad Sci USA* **91**: 2315–2319

Buscial L, Esteve J-P, Saint-Laurent N, Bertrand V, Reisine T, O'Carrol A, Bell GI, Schally AV, Vaysse N and Susini N (1995) Inhibition of cell proliferation by the somatostatin analogue RC-160 is mediated by somatostatin receptor subtypes SSTR2 and SSTR5 through different mechanisms. *Proc Natl Acad Sci USA* **92**: 1580–1584

Cancer Research Campaign factsheet 1 (1990) Cancer Research Campaign, UK.

Cancer Research Campaign factsheet 18 (1993) Cancer Research Campaign, UK.

Cascinu S, Del Ferro E and Catalano G (1995) A randomised trial of octreotide versus best supportive care only in advanced gastrointestinal cancer patients refractory to chemotherapy. *Br J Cancer* **71**: 97–101

Eden PA and Taylor JE (1993) Somatostatin receptor subtype gene expression in human and rodent tumors. *Life Sci* **53**: 85–90

Klijn JGM, Hoff AM, Planting AST Verweij J, Kok T, Lamberts SWJ, Portengen H and Foekens JA (1990) Treatment of patients with metastatic pancreatic and gastrointestinal tumors with the somatostatin analogue Sandostatin: a phase II study including endocrine effects. *Br J Cancer* **62**: 627–630

Krook J, Goldberg RM, Moertel CG and Wieand HS (1993) A phase III evaluation of the somatostatin analogue octreotide in the therapy of assymptomatic advanced colon cancer: a North Central Cancer Treatment Group Study. *Proc A SCO* **12**: 191

Lamberts SWJ, Van Der Lely A-J, De Herder WW and Hofland LJ (1996) Octreotide. *N Engl J Med* **334**: 246–253

Liebow C, Reilly C, Serrono M and Schally AV (1989) Somatostatin analogues inhibit growth of pancreatic cancer by stimulating tyrosine phosphatase. *Proc Natl Acad Sci USA* **86**: 2003–2007

Miller GV, Farmery SM, Woodhouse L F and Primrose JN (1992) Somatostatin binding in normal and malignant human gastrointestinal mucosa. *Br J Cancer* **66**: 391–395

NIH Consensus Conference (1990) Adjuvant therapy for patients with colon and rectal cancer. *JAMA* **264**: 1444–1450

O'Carroll A, Lolait S, Konig M and Mahan LC (1993) Molecular cloning and expression of a pituitary somatostatin receptor with preferential affinity for somatostatin-28. *Mol Pharmacol* **42**: 939–946

O'Carroll A, Raynor K, Lolait SJ and Reisine T (1994) Characterisation of cloned human somatostatin receptor SSTR5. *Mol Pharmacol* **46**: 291–298

Pagliacci MC, Tognellini R, Grignani F and Nicoletti I (1991) Inhibition of human breast cancer cell (MCF-7) growth *in vitro* by the somatostatin analog SMS 201–995: effects on cell cycle parameters and apoptotic cell death. *Endocrinology* **129**: 2555–2562

Palmer Smith J, Doll D, Croitoru R, Thornton C and Perry MC (1994) Octreotide has no effect on advanced colon cancer. *J Clin Gastroenterol* **18**: 245–247

Partsch G and Matucci-cerinic M (1992) Effect of substance P and somatostatin on migration of polymorphonuclear (PMN) cells *in vitro*. Inflammation **16**: 539–547

Patel YC, Zing HH, Fitz-Patrick D and Srikant CB (1981) Somatostatin: some aspects of its physiology and pathophysiology. In *Gut Hormones*, Bloom S R and Polak J M (eds), pp. 339–349. Churchill Livingstone: Edinburgh

Patel YC, Greenwood MT, Warszynska A, Panetta R and Srikant CB (1994) All five cloned human somatostatin receptors (hSSTR1-5) are functionally coupled to adenyl cycalse. *Biochem Biophys Res Commun* **193**: 605–612

Pinski J, Halmos G, Yano T, Szepeshazi K, Qin Y, Ertl T and Schally AV (1994) Inhibition of growth of MKN45 human gastric carcinoma xenografts in nude mice by treatment with bombesin/gastrin releasing peptide antagonist (RC-3095) and somatostatin analogue (RC-160). *Int J Cancer* **57**: 574–580

Qin Y, Schally AV and Willems G (1992) Treatment of liver metastases of human colon cancer in nude mice with somatostatin analogue RC-160. *Int J Cancer* **52**: 791–796

Radulovic S, Comaru-Schally AM, Milovanovic S and Schally AV (1993) Somatostatin analogue RC-160 and LHRH antagonist SB-75 inhibit growth of MIA PaCa-2 human pancreatic xenografts in nude mice. *Pancreas* **8**: 88–97

Raynor K, Murphy WA, Coy DH, Taylor JE, Moreau J-P, Yasuda K, Bell GI and Reisine T (1993) Cloned somatostatin receptors: identification of subtype selective peptides and demonstration of high affinity binding of linear peptides. *Mol Pharmacol* **43**: 838–844

Reubi JC, Horisberger U and Laissue J (1994) High density of somatostatin receptors in veins surrounding human cancer tissue: role in host – tumor interaction? *Int J Cancer* **56**: 681–688

Rohrer L, Raulf F, Bruns C, Buettner R, Hofstaedter F and Schule R (1993) Cloning and characterisation of a fourth human somatostatin receptor. *Proc Natl Acad Sci USA* **90**: 4196–4200

Singh P, Le S, Townsend CM, Beauchamp RD, Laridjani A and Thompson JC (1986) A long acting somatostatin analogue (SRIF)(SMS201-995) and proglumide (PGL) inhibit the trophic and gastrin receptor (GR) regulatory effects of pentagastrin (PG) on mouse colon cancer cells (MC-26) *in vivo* (abstract). *Gastroenterology* **90**: 1636

Stewart GJ, Lawson JA and Morris DL (1994) Octreotide inhibits development of hepatic metastases from a human colonic cancer cell line. *Br J Surg* **81**: 1332

Todisco A, Seva C, Dickinson C J and Yamada T (1994) Somatostatin inhibits AP-1 function via multiple protein phosphatases. *Gastroenterology* **106**: A846

van Eijck CHJ, Slooter GD, Hofland LJ, Kort W, Jeekel J, Lamberts SWJ and Marquet RL (1994) Somatostatin receptor dependent growth inhibition of liver metastases by octreotide. *Br J Surg* **81**: 1333–1337

Vidal C, Rauly I, Zeggari M, Delesque N, Esteve JP, Saint- Laurent N, Vaysse N and Susini C (1994) Up-regulation of somatostatin receptors by epidermal growth factor and gastrin in pancreatic cancer cells. *Mol Pharmacol* **45**: 97–104

Warhurst G, Higgs NB, Grigor MR, Ross I and Barbezat GO (1995) Expression of multiple somatostatin receptor genes in human colonic epithelial cells. *Biochem Soc Trans* **23**: 18s

Woltering EA, Barrie R, O'Dorisio TM, Acre D, Ure T, Cramer A, Holmes D, Robertson J and Fassler J (1990) Somatostatin analogues inhibit angiogenesis in the chick chorioallantoic membrane. *Digestion* **46**(suppl.): 343

Yamada Y, Post SR, Wang K, Tager HS, Bell GI and Seino S (1992*a*) Cloning an functional characterisation of a family of human and mouse somatostatin receptors expressed in brain, gastrointestinal tract and kidney. *Proc Natl Acad Sci USA* **89**: 251–255

Yamada Y, Reisine T, Law SF, Ihara Y, Kubota A, Kagimoto S, Seino M, Seino Y, Bell GI and Seino S (1992*b*) Somatostatin receptors, an expanding gene family: cloning and functional characterisation of human SSTR3, a protein coupled to adenyl cyclase. *Mol Endocrinol* **6**: 2136–2142

Yamada Y, Kagimoto S, Kubota A, Yashuda K, Masuda K, Someya Y, Ihara Y, Li Q, Imura H, Seino S and Seino Y (1993) Cloning, functional expression and pharmacological characterisation of a fouth (hSSTR4) and fifth (hSSTR5) somatostatin receptor subtype. *Biochem Biophy Res Commun* **195**: 844–852

© Cancer Research Campaign 1997